# Asymptomatic Pulmonary Edema in Children With Secundum Atrial Septal Defect: A Bedside Lung Ultrasound Screening Study

**DOI:** 10.7759/cureus.112630

**Published:** 2026-07-14

**Authors:** Ali Alharbi, Maryam Alkhalaf, Noha Alraddadi, Omar Tamimi

**Affiliations:** 1 Pediatric Cardiac Intensive Care, Prince Sultan Cardiac Center, Riyadh, SAU; 2 Pediatric Cardiology, King Salman Heart Center, King Fahad Medical City, Riyadh, SAU

**Keywords:** b-line, children, congenital heart disease, lung ultrasound, pulmonary edema, secundum atrial septal defect

## Abstract

Background: Secundum atrial septal defect (ASD II) is a common congenital heart defect that produces a left-to-right shunt and increases pulmonary blood flow. Whether chronic pulmonary overcirculation raises extravascular pulmonary fluid before overt symptoms appear is uncertain. Lung ultrasound is a rapid, noninvasive, radiation-free bedside method that detects extravascular lung fluid through B-line artifacts and is an established tool for assessing pulmonary congestion.

Objective: This study aimed to screen for sonographic evidence of pulmonary edema in clinically asymptomatic children with isolated ASD II using bedside lung ultrasound.

Methods: This prospective observational single-center study was conducted from January 2025 to May 2026. Children aged two to eight years with an echocardiographically confirmed ASD II, without chronic lung disease and without another significant shunt lesion or complex congenital anatomy, were screened. Echocardiography was performed per American Society of Echocardiography guidelines, and the pulmonary-to-systemic flow ratio (Qp/Qs) was estimated echocardiographically. Lung ultrasound was performed at the bedside with a portable system (SonoSite; Fujifilm SonoSite Inc., Bothell, WA) using a high-frequency hockey-stick linear probe (HSL25x, 6-13 MHz). A scan was defined as positive when at least three B-lines were present in at least two bilateral lung zones. Descriptive statistics were used.

Results: Ten children were included (seven female, three male; mean age, 4.3 years; range, 2-8). Mean ASD diameter was 16.2 mm (range, 9.6-28.0), and the mean Qp/Qs was 2.8 (range, 1.26-4.4). Pro-B-type natriuretic peptide was available in eight of 10 children and was within the normal range in all of them. Lung ultrasound was positive in one of 10 children (10%) and negative in nine (90%). The single positive scan occurred in a child with a normal natriuretic peptide level, no comorbidity, and a moderate defect; the children with the largest defect and the highest shunt ratios all had negative scans.

Conclusions: In this small cohort of asymptomatic children with isolated ASD II, sonographic signs of increased extravascular lung water were uncommon and did not correlate with defect size, shunt magnitude, or natriuretic peptide level. Bedside lung ultrasound was feasible. These findings require confirmation in larger, controlled studies.

## Introduction

Atrial septal defect (ASD) is the most frequent form of congenital heart disease, and the secundum subtype (ASD II), arising from a deficiency of the fossa ovalis, accounts for the majority of atrial-level communications. Regional studies from Saudi Arabia and Egypt confirm that ASD II is a common contributor to the overall burden of congenital heart disease in children [1-4].

An ASD II creates a left-to-right shunt whose magnitude depends on defect size and the relative compliance of the ventricles. The resulting increase in pulmonary blood flow, expressed as the pulmonary-to-systemic flow ratio (Qp/Qs), produces chronic volume loading of the right heart and the pulmonary vascular bed. Although many patients remain asymptomatic for years and the lesion is often first detected through an incidental murmur [5], sustained pulmonary overcirculation is the physiological basis for the right-heart dilation, exercise limitation, arrhythmia, and pulmonary vascular disease that may develop over time.

A question that follows from this physiology is whether chronic pulmonary overcirculation produces a measurable increase in extravascular lung fluid before the child becomes clinically symptomatic. Traditional bedside assessment, auscultation, oxygen saturation, and chest radiography are relatively insensitive to small changes in lung fluid. Natriuretic peptides, such as pro-B-type natriuretic peptide, provide a circulating marker of myocardial wall stress but are influenced by age, renal function, and comorbidity, and do not directly image the lung [6].

Lung ultrasound has emerged as a sensitive, reproducible, and entirely noninvasive means of detecting extravascular lung fluid. Vertical reverberation artifacts known as B-lines increase in number as lung fluid accumulates, and the technique has been validated against other measures of pulmonary congestion in adults [7,8] and compared favorably with chest radiography for fluid overload in critically ill children [9]. Its portability, absence of radiation, and suitability for repeated bedside use make it attractive for pediatric cardiology.

To our knowledge, there is no study examining whether asymptomatic children with an isolated ASD II exhibit sonographic evidence of subclinical pulmonary edema and whether any such finding relates to defect size, shunt ratio, or natriuretic peptide levels. This study was designed to address that gap by systematically applying bedside lung ultrasound to a cohort of children with isolated ASD II and relating the findings to their hemodynamic and clinical profile.

## Materials and methods

Study design and setting

This was a prospective observational single-center study carried out from January 2025 to May 2026. Consecutive eligible children with ASD II were screened with bedside lung ultrasound and characterized clinically and echocardiographically.

Participants

Eligible children were identified from those undergoing echocardiographic evaluation. The inclusion and exclusion criteria were as follows. The inclusion criteria were children aged two to eight years, an ASD II lesion confirmed on echocardiography, and no chronic lung disease or other significant congenital heart defect. The exclusion criteria were a significant additional heart defect with a shunt or complex congenital cardiac anatomy.

Echocardiography

The diagnosis of ASD II was established by transthoracic echocardiography performed in accordance with the American Society of Echocardiography guidelines for a comprehensive pediatric transthoracic echocardiogram [10]. The maximum defect diameter (millimeter) was recorded, and the Qp/Qs was estimated echocardiographically. Children with an additional significant shunt lesion or complex anatomy were excluded as above.

Lung ultrasound

Lung ultrasound was performed at the bedside using a portable ultrasound system (SonoSite; Fujifilm SonoSite Inc., Bothell, WA) with a high-frequency hockey-stick linear transducer (HSL25x, 6-13 MHz). Scanning followed the approach described by Picano et al. for lung ultrasound in cardiology [7]. Lung zones were examined bilaterally, and the presence and number of B-lines, and vertical laser-like reverberation artifacts arising from the pleural line were recorded together with the pleural-line appearance and the underlying A-line/B-line pattern [7,8]. The probe selection followed established lung ultrasound recommendations [11].

A lung ultrasound scan was defined as positive when at least three B-lines were present in at least two bilateral lung zones. Scans not meeting this threshold were classified as negative.

Data collected

For each child, the following were recorded: sex, age, height and weight with percentiles, body surface area, ASD diameter, Qp/Qs, comorbidities, pro-B-type natriuretic peptide, the lung ultrasound result (positive or negative), and a descriptive comment on the sonographic pattern.

Statistical analysis

Given the small sample size, only descriptive statistics were used. Continuous variables are summarized as mean, median, range, and standard deviation; categorical variables as counts and percentages. A descriptive comparison between the positive and negative lung ultrasound groups is presented, but formal hypothesis testing was not performed because the number of positive scans (n = 1) is far too small to support valid inferential statistics.

Ethical considerations

The study was reviewed and approved by the Institutional Review Board of King Fahad Medical City (Riyadh Second Health Cluster), Riyadh, Saudi Arabia (IRB log number 24-432; category: exempt; approved August 11, 2024), in accordance with International Conference on Harmonisation Good Clinical Practice guidelines.

## Results

Cohort characteristics

Ten children were screened and included. Seven (70%) were female and three (30%) were male patients. The mean age was 4.3 years (median, 4.0; range, 2-8; standard deviation (SD), 1.9). The mean echocardiographic ASD diameter was 16.2 mm (median, 16.0; range, 9.6-28.0; SD, 5.2), and the mean Qp/Qs was 2.8 (median, 3.0; range, 1.26-4.4; SD, 1.0), indicating a predominantly moderate-to-large left-to-right shunt across the cohort. The baseline characteristics are summarized in Table 1.

**Table 1:** Baseline demographic and clinical characteristics of the cohort (n = 10) ASD: atrial septal defect; Qp/Qs: pulmonary-to-systemic flow ratio; pro-BNP: pro-B-type natriuretic peptide

Characteristic	Value
Number of children	10
Female, n (%)	7 (70%)
Male, n (%)	3 (30%)
Age (years), mean (median; range)	4.3 (4.0; 2-8)
ASD diameter (mm), mean (median; range)	16.2 (16.0; 9.6-28.0)
Qp/Qs, mean (median; range)	2.8 (3.0; 1.26-4.4)
Pro-BNP available, n	8 of 10
Pro-BNP within normal range, n	8 of 8
Any comorbidity, n (%)	5 (50%)
Down syndrome, n	2
Bronchial asthma, n	2
No comorbidity, n (%)	5 (50%)
Lung ultrasound positive, n (%)	1 (10%)
Lung ultrasound negative, n (%)	9 (90%)

Comorbidities were present in five of 10 children (50%). Two children had Down syndrome (one with coexisting cleft palate and one with congenital hypothyroidism), two had bronchial asthma, one had a confirmed ARID1A mutation consistent with Coffin-Siris syndrome, and one had dysmorphic features with a negative whole-exome sequencing result. Five children (50%) had no recorded comorbidity. Pro-B-type natriuretic peptide was available in eight of 10 children, and all eight available values were within the laboratory normal range (below 300 pg/dL).

Lung ultrasound findings

Lung ultrasound was positive in one of 10 children (10%) and negative in nine (90%). Across the negative scans, the dominant appearance was an A-line pattern, with several children accompanied by a small number of simple (isolated) B-lines in the lower zones, an appearance generally regarded as within normal limits. The individual findings are presented in Table 2.

**Table 2:** Individual patient findings N: within normal range; NA: not available; LUS: lung ultrasound; pro-BNP: pro-B-type natriuretic peptide; ASD: atrial septal defect; Qp/Qs: pulmonary-to-systemic flow ratio

S. no.	Age	Sex	ASD (mm)	Qp/Qs	Pro-BNP (pg/dL)	LUS result
1	4 years	M	18.3	1.8	44 (N)	Negative
2	3 years	M	14	3.0	NA	Negative
3	4 years	F	18	1.6	131 (N)	Negative
4	8 years	F	28	3.0	33 (N)	Negative
5	2 years	F	11	3.0	130 (N)	Negative
6	7 years	F	18	4.4	91 (N)	Negative
7	2 years	F	9.6	2.3	NA	Negative
8	4 years	F	13	3.2	115 (N)	Positive
9	4 years	M	14	1.26	50 (N)	Negative
10	5 years	F	18	4.0	139 (N)	Negative

The single positive scan occurred in a four-year-old girl with a 13-mm defect and Qp/Qs 3.2 and no comorbidity, whose scan showed confluent B-lines in the right lower zone; her pro-B-type natriuretic peptide was within the normal range (115). Notably, the child with the largest defect (28 mm) and the children with the highest shunt ratios (Qp/Qs 4.4:1) all had negative scans, so positivity did not align with the greatest hemodynamic burden in this cohort. A descriptive comparison of the positive and negative scans is shown in Table 3.

**Table 3:** Descriptive comparison of positive vs. negative lung ultrasound scans ASD: atrial septal defect; Qp/Qs: pulmonary-to-systemic flow ratio; pro-BNP: pro-B-type natriuretic peptide

Variable	Positive (n = 1)	Negative (n = 9)
Female, n	1 of 1	6 of 9
Age (years), mean (range)	4.0	4.3 (2-8)
ASD diameter (mm), mean (range)	13.0	16.5 (9.6-28.0)
Qp/Qs, mean (range)	3.2	2.7 (1.26-4.4)
Pro-BNP within normal range	1 of 1	7 of 7 available
Any comorbidity, n	0 of 1	5 of 9

## Discussion

In this prospective cohort of 10 asymptomatic children with isolated ASD II, bedside lung ultrasound was positive for increased extravascular lung fluid in only one child (10%). Despite a cohort-wide tendency toward moderate-to-large shunts (mean Qp/Qs, 2.8), sonographic evidence of pulmonary edema was the exception rather than the rule, and the negative scans were dominated by a normal A-line pattern. All available natriuretic peptide values were within the normal range.

The central observation is that subclinical pulmonary edema was uncommon in these asymptomatic children and, where present, did not align with the markers of greatest hemodynamic burden. The child with the largest defect (28 mm) and those with the highest shunt ratios (Qp/Qs, 4.4 and 4.0) all had negative scans, and the single positive scan occurred in a child with only a moderate defect. This dissociation argues against a simple, direct relationship between shunt magnitude and measurable pulmonary extravascular fluid in this age group, and it is physiologically plausible: in an isolated ASD, the volume load falls primarily on the right heart and the low-pressure pulmonary circulation, and the pulmonary lymphatics and a compliant pulmonary bed may accommodate increased flow without a rise in extravascular lung fluid.

The single positive scan is also notable for occurring with a normal natriuretic peptide level and no comorbidity, which leaves its clinical significance uncertain in isolation. Across the cohort, the uniformly normal natriuretic peptide values are consistent with the largely asymptomatic clinical status.

Lung ultrasound is a validated tool for detecting extravascular pulmonary fluid and assessing pulmonary congestion in cardiology, with B-lines serving as the key sonographic marker [7,8]. In the pediatric critical-care setting, lung ultrasound has been compared favorably with chest radiography for detecting fluid overload, supporting its feasibility and sensitivity in children [9]. The natural history and natriuretic-peptide literature in ASD indicate that the hemodynamic consequences of an atrial-level shunt, including effects on growth and on serum natriuretic peptides, evolve and are modified by treatment [6,12], which is consistent with the normal natriuretic peptide values and the infrequent positive scan observed here in young, asymptomatic patients. Regional epidemiological data confirm that ASD is a common lesion in the populations from which such cohorts are drawn [1,3,4]. The present study extends this body of work by applying lung ultrasound specifically to screen asymptomatic children with isolated ASD II, a question not directly addressed by the available literature.

Clinical implications

Bedside lung ultrasound was feasible, quick, and radiation-free in this pediatric cardiology setting and reliably produced an interpretable A-line/B-line pattern. The low percentile of positive cases without symptoms may add another perspective to check for symptomatic patients with ASD II before considering medical management.

Limitations

The small sample size, with only 10 children and a single positive scan, makes the study underpowered for any inferential analysis; all results are descriptive and may be affected by chance. As a single-center design, Findings may not generalize to other settings, populations, or scanning protocols. The confounding comorbidities, Bronchial asthma, Down syndrome, and genetic syndromes, may affect the lung scan and pro-B-type natriuretic peptide.

Future directions

Larger, multicenter studies with a matched control group, blinded interpretation, and longitudinal follow-up are needed to determine whether lung ultrasound has a role in monitoring children with ASD II and whether any sonographic signal predicts clinically relevant outcomes.

## Conclusions

In this small prospective cohort of asymptomatic children with isolated secundum ASD, sonographic evidence of increased extravascular lung fluid was uncommon (one of 10) and did not correspond to defect size, shunt ratio, or natriuretic peptide level, all of which were normal. A bedside lung ultrasound was feasible and well tolerated. These preliminary findings should not be overinterpreted and warrant confirmation in larger, controlled studies before lung ultrasound is adopted for routine pulmonary congestion screening in this population.
